# Low Serum 25-Hydroxyvitamin D Concentrations Are Associated with Increased Risk for Melanoma and Unfavourable Prognosis

**DOI:** 10.1371/journal.pone.0112863

**Published:** 2014-12-01

**Authors:** Benjamin Bade, Alexander Zdebik, Stefan Wagenpfeil, Stefan Gräber, Jürgen Geisel, Thomas Vogt, Jörg Reichrath

**Affiliations:** 1 Center for Clinical and Experimental Photo-Dermatology, Department of Dermatology, The Saarland University Hospital, Homburg, Germany; 2 Institute of Medical Biometry, Epidemiology and Medical Informatics, The Saarland University Hospital, Homburg, Germany; 3 Department of Clinical Chemistry and Laboratory Medicine, The Saarland University Hospital, Homburg, Germany; Duke University Medical Center, United States of America

## Abstract

**Background:**

Low vitamin D status (serum 25(OH)D concentration) is associated with increased incidence and unfavourable outcome of various types of cancer. However, there are limited data on influence of serum 25(OH)D on risk and prognosis of malignant melanoma.

**Methods:**

Basal serum 25(OH)D concentrations were retrospectively analyzed in a cohort of melanoma patients (n = 324) and healthy controls (n = 141). We tested the hypothesis that serum 25(OH)D concentrations are predictive of melanoma risk, thickness of primary melanomas, and overall survival (OS).

**Results:**

Median serum 25(OH)D concentrations were significantly lower (p = 0.004) in melanoma patients (median = 13.6 ng/ml) as compared to controls (median = 15.6 ng/ml). Primary tumors of patients with low serum 25(OH)D concentrations (<10 ng/ml) had significantly (p = 0.006) greater Breslow thickness (median: 1.9 mm) as compared to patients with higher levels (>20 ng/ml; median: 1.00 mm). Patients with 25(OH)D serum concentrations in the lowest quartile had inferior overall survival (median: 80 months) comparing with the highest quartile (median: 195 months; p = 0.049).

**Conclusions:**

Our data support the concept that serum 25(OH)D concentrations are associated with risk and prognosis of melanoma. Whether normalizing serum 25(OH)D concentrations in these patients improves outcomes will require testing in future clinical trials.

## Introduction

Over the last 20 years, an increasing body of epidemiological and experimental studies has supported the so called “vitamin D/cancer hypothesis”. The basis of the experimental support was the demonstration of the almost ubiquitous expression of both the vitamin D receptor (VDR) [1], [2] and the activating enzyme (vitamin D-1α-hydroxylase, CYP27B1) [3], which converts serum 25-hydroxyvitamin D_3_ (25(OH)D) into the biologically active vitamin D metabolite 1,25-dihydroxyvitamin D_3_ (1,25(OH)_2_D). Binding and transcriptional activation of VDR by 1,25(OH)_2_D regulate multiple cellular functions that are implicated in carcinogenesis and cancer progression, including proliferation, differentiation, apoptosis, angiogenesis and metastatic potential [4]–[10]. In line with these experimental studies, numerous pro- and retrospective epidemiological studies have now convincingly shown that low serum 25(OH)D concentrations are associated in colorectal cancer and various other types of cancer with a significantly increased risk for the development of the disease [11]–[16] and a relatively poor prognosis [17]–[19]. In agreement with these findings, a meta-analysis of five epidemiological studies showed a 51% reduction for the risk of colorectal cancer in people with serum 25(OH)D concentrations in the highest versus the lowest quintiles (p<0.0001) [20]. Additionally, a prospective, randomised, controlled trial (RCT) of vitamin D and calcium supplementation in 1,179 women revealed a 60% reduction in all-cancer risk in the intervention arm (p<0.03) [21]. It has to be noted that the majority of RCTs published so far, analyzing the impact of vitamin D on cancer, showed no significant results. However, most of these studies were limited due to poor study designs, including low doses and/or short duration of the supplementation with vitamin D, or because of low compliance [22], [23].

Meanwhile, it is well accepted that lack of sun exposure leads to vitamin D deficiency, since 90% of all gained vitamin D has to be formed in the skin by UV-B exposure [24]–[25]. The impact of solar UV-exposure on incidence and prognosis of melanoma is yet not well understood, as is the influence of the cutaneous synthesis of vitamin D [26]–[27]. On one side, increased risk of melanoma is reported in individuals with a history of recurring intensive, short time UV exposure (most importantly sunburns in childhood), especially in fair-skinned individuals [28]–[29]. On the other side, moderate sun exposure has been reported to exert a protective effect against and to be associated with a relatively favourable prognosis and increased overall survival rates in melanoma patients [26], [27], [30]. Additionally, some pilot studies have supported the hypothesis that serum 25(OH)D concentrations may be associated with risk and prognosis of melanoma [31], [32]. To gain further insights into the relevance of serum 25(OH)D concentrations on risk and prognosis of melanoma, we have now carried out this retrospective study in 324 German patients and 141 healthy controls.

## Methods

This retrospective study was performed in agreement with the Ethical committee of the “Ärztekammer des Saarlandes” (No. 247/12). Patient records/information was anonymized and de-identified prior to analysis. The study population included 324 Patients from the Department of Dermatology of the University of Regensburg with histologically proven primary cutaneous melanomas of different stages. All 324 venous blood samples were taken between January 2000 and December 2004. In 291 melanoma patients, blood samples were taken at the time of primary diagnosis. 33 melanoma patients were diagnosed between 1984 and 1999 (venous blood samples were taken between January 2000 and December 2004 in these patients as well). All blood samples were immediately processed and separated, then aliquoted and stored at −40°C. Basal 25(OH)D serum concentrations (ng/ml; 1 ng/ml = 2.5 nmol/L) were analyzed at the Department of Clinical Chemistry and Laboratory Medicine of the Saarland University Hospital in Homburg using LIAISON® 25-OH Vitamin D Total (DiaSorin, Dietzenbach, Germany). The lower detection limit of this assay is at 4 ng/ml, and the within-pair coefficient of variation (CV) of this assay was in a recently published study 4.9% using blinded quality control samples [33].

Clinical data were abstracted from medical records using a standard protocol. The observation time of this retrospective study started at the time of diagnosis, patients were observed until death or December 2004, whichever came first. Venous blood samples from the control group (141 healthy German individuals; 60 men, 81 women; age: mean 55.09, median 54, 14–34 years: n = 40, 35–64 years: n = 66, >/ = 65 years: n = 35) were collected at the Saarland University Hospital between February 2001 and April 2006, data have been published previously [31].

The study population was divided into different groups of interest (e.g. 25(OH)D levels, tumor thickness, survival, gender, age, time of venipuncture); medians, means and standard deviations were calculated. Because 25(OH)D serum concentrations could not be assumed to be normally distributed (demonstrated by Kolmogorov-Smirnov-Test), differences between individual groups were analysed with the Mann-Whitney U-test, Kruskal-Wallis-test, or with Log-Rank-test in Kaplan-Meier analyses. A p-value of <0.05 was considered statistically significant. Statistical analysis also included multivariate analysis and ANOVA. All statistical analyses were performed using the statistical package SPSS Version 18 (IBM, USA).

## Results

### Moderately lower serum 25(OH)D concentrations in melanoma patients (diagnosed in wintertime) as compared to controls

Population characteristics is displayed in Table 1. Blood samples of the control group and from melanoma patients were matched by season. For blood samples of the control group were taken from Fall through Spring (Oct-Apr), they were compared with a cohort of melanoma patients where blood was taken in the same period (Oct-Apr). In both study groups, about 63% of the population had a deficient vitamin D status (serum 25(OH)D levels <20 ng/ml). Median serum 25(OH)D concentrations were significantly lower (p = 0.004) in melanoma patients (median = 13.6 ng/ml; n = 127) as compared to controls (median = 15.6 ng/ml; n = 141) (Table 1). Additional statistical tests, including age and time of blood draw (BMI data were not available) in multiple analyses and ANOVA, confirmed our results. Considering 25(OH)D as dependent and sex, age, season of blood draw as well as group as independent variables, the adjusted group effect resulted in a p-value of 0,012 (Table 2).

**Table 1:** Please note lower serum 25(OH)D concentrations in melanoma patients (diagnosed in wintertime) as compared to controls, and in metastasized as compared to non-metastasized patients.

	Melanoma patients	Control	Total
Variable	(n = 324)	(n = 141)	(n = 465)
Gender			
Men	174	60	234
Women	150	81	231
Age			
Mean	56.3	55.1	
Median	60.0	54.0	
<51	105	84	189
Serum 25(OH)D median	18.5 ng/ml	13.85 ng/ml	
52–66	108	27	135
Serum 25(OH)D median	16.4 ng/ml	18.4 ng/ml	
>67	111	30	141
Serum 25(OH)D median	14.2 ng/ml	16.25 ng/ml	
	p = 0.003	p = 0.38	
Serum 25(OH)D (ng/ml)			
n	324	141	465
Mean	18	18,77	18,23
Median	16,2	15,6	15,7
SD	10,59	11,11	10,74
Interquatile range	14.5 ng/ml	17.2 ng/ml	14.3 ng/ml
Serum 25(OH)D (grouping)			
<20 ng/ml	207 (63.89%)	89 (63.1%)	296 (63.7%)
<10 ng/ml	82 (39.6%)	39 (43.8%)	121 (40.9%)
10–20 ng/ml	125 (60.4%)	50 (56.2%)	175 (59.1%)
>20 ng/ml	117 (36.11%)	52 (36.9%)	169 (36.3%)
Vitamin D status in melanoma patients vs. Controls (venipunctured in winter)			
	all MMs	Control	
n	127	141	268
Mean	15,1	18,8	
Median	13,6	15,6	
SD	10,1	11,1	
p = 0.004			
Serum 25(OH)D levels (ng/ml) in metastasized and non-metastasized melanoma patients			
	metastasized	non-metastasized	
n	55	269	324
Mean	16,63	18,29	
Median	15,3	16,2	
SD	10,67	10,57	
p = 0.29			

In the cohort of all melanoma patients significant (p = 0.003) age-related differences in serum 25(OH)D concentrations were observed (20–51 yrs.: median = 18.5 ng/ml, n = 105; 52–66 yrs.: median = 16.4 ng/ml, n = 108; >67 yrs.: median = 14.2 ng/ml, n = 111).

**Table 2:** Multiple analyses confirm that median serum 25(OH)D concentrations are significantly lower in melanoma patients as compared to controls.

Dependent variable: serum 25(OH)D concentration		
	Regression coefficient	p-value
Independent variables:		
Gender	−0.41	0.67
Season of blood draw	4.7	0.00
Age	−0.09	0.002
Group	3.1	0.012

Additional statistical tests, including age and time of blood draw (BMI data were not available) in multiple analyses and ANOVA, confirmed that median serum 25(OH)D concentrations are significantly lower in melanoma patients as compared to controls. Considering 25(OH)D as dependent and sex, age, season of blood draw as well as group as independent variables, the adjusted group effect resulted in a p-value of 0,012.

### Serum 25(OH)D concentrations are associated with thickness of primary melanomas

**Figure 1:**
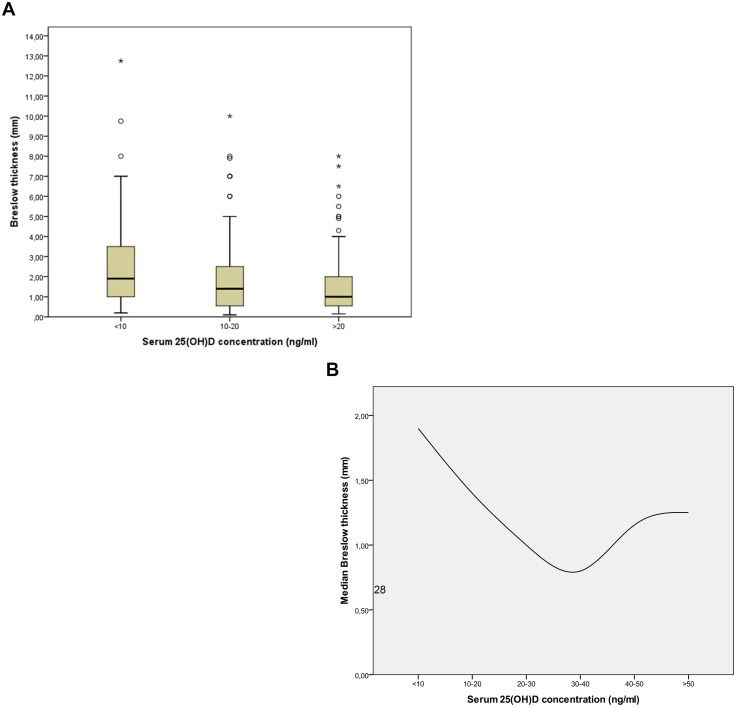
Serum 25(OH)D concentrations are associated with Breslow thickness of primary melanomas. Median Breslow thickness (black horizontal line) in melanoma patients is shown (1a) (<10 ng/ml = 1.9 mm, n = 77; 10–20 ng/ml = 1.4 mm, n = 122; >20 ng/ml = 1 mm, n = 108; total n = 307; p = 0.002). 17 Patients were excluded from this cohort due to missing clinical data. Boxes represent the values within the 25th and 75th percentiles (1a). We report evidence of an inverse J-shaped curve (p = 0.033), indicating that high serum 25(OH)D concentrations (>40 ng/ml) may be associated with thicker primary melanomas as well. Median Breslow thickness and 25(OH)D serum concentration at time of diagnosis from melanoma patients is shown (<10 ng/ml = 1.9 mm [ci 95%: 1.3–2.5], n = 77; 10–20 ng/ml = 1.4 mm [ci 95%: 1.1–1.8], n = 122; 20–30 ng/ml = 1.00 mm [ci 95%: 0.73–1.5], n = 65; 30–40 ng/ml = 0.8 mm [ci 95%: 0.4–1.85], n = 31; 40–50 ng/ml = 1.155 mm [ci 95%: 0.65–2.7], n = 8; >50 ng/ml = 1.25 mm [ci 95%: 0.5–2.0], n = 4; total n = 307; p = 0.033). 17 Patients were excluded from the cohort due to missing clinical data (1b).

Patients with serum 25(OH)D concentrations <20 ng/ml (n = 199; median = 1.53 mm) had significantly (p = 0.002) thicker primary melanomas than patients with concentrations >20 ng/ml (n = 108; median = 1.00 mm) (Fig. 1a). Furthermore, we here report for the first time evidence of an inverse J-shaped curve (p = 0.033), indicating that high serum 25(OH)D concentrations (>40 ng/ml; n = 12; median = 1.155 mm) maybe associated with thicker primary melanomas as well (Fig. 1b). Additional statistical tests, including gender and time of blood draw in multiple analyses, confirmed our finding that serum 25(OH)D concentrations are associated with Breslow thickness of primary melanomas (n = 307, p-value = 0.013) (Table 3).

**Table 3:** Multiple analyses confirm the association of serum 25(OH)D concentration with Breslow thickness of primary melanomas.

Dependent variable: Breslow thickness of primary melanomas		
	Regression coefficient	p-value
Independent variables:		
Gender	0.23	0.31
Season of blood draw	−0.04	0.86
serum 25(OH)D concentration	−0.026	0.013

Additional statistical tests, including gender and time of blood draw in multiple analyses, confirmed our finding that serum 25(OH)D concentrations are associated with Breslow thickness of primary melanomas (n = 307; 17 Patients were excluded from this cohort due to missing clinical data). Considering Breslow thickness of primary melanomas as dependent and gender, season of blood draw as well as serum 25(OH)D concentration as independent variables, the adjusted serum 25(OH)D concentration effect resulted in a p-value of 0.013.

### In contrast to serum 25(OH)D concentration, Breslow thickness of primary melanomas is not associated with season

**Figure 2:**
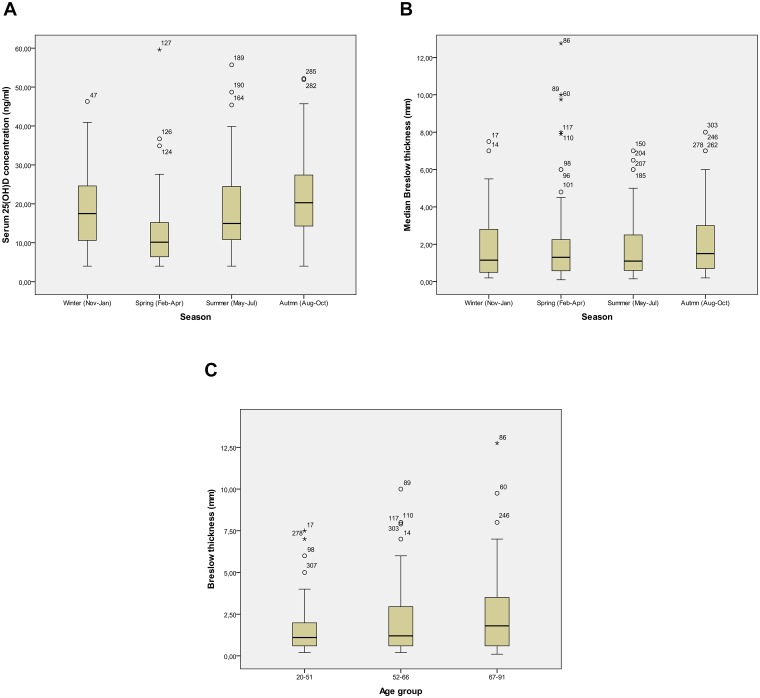
25(OH)D concentration, age, season and Breslow thickness of primary melanomas. We found significant (p<0.001) variations in serum 25(OH)D concentrations throughout the seasons in melanoma patients (n = 324), with a peak in autumn (Aug-Oct) (median = 21.3 ng/ml) and a low in springtime (Feb-Apr) (median = 10.15 ng/ml) (2a). An association of Breslow thickness and season was not observed (p = 0·543, n = 307), 17 patients from the cohort were excluded due to missing clinical data (2b). Additionally, we found (p = 0.07) age-related differences in Breslow thickness of primary melanomas (20–51 yrs.: median = 1.1 mm; 52–66 yrs.: median = 1.2 mm; >67 yrs.: median = 1.8 mm) (2c). Boxes represent the values (median) within the 25^th^ and 75^th^ percentiles.

We found significant (p = 0.000) variations in serum 25(OH)D concentrations throughout the seasons in melanoma patients (n = 324), with a peak in autumn (Aug–Oct) (median = 21.3 ng/ml) and a low in springtime (Feb–Apr) (median = 10.15 ng/ml) (Fig. 2a). An association of Breslow thickness and season was not observed (p = 0.543) (Fig. 2b).

### Age is associated with serum 25(OH)D concentrations and with Breslow thickness of primary melanomas

In the cohort of all melanoma patients significant (p = 0.003) age-related differences in serum 25(OH)D concentrations were observed (20–51 yrs.: median = 18.5 ng/ml, n = 105; 52–66 yrs.: median = 16.4 ng/ml, n = 108; >67 yrs.: median = 14.2 ng/ml, n = 111) (Table 1). Additionally, we found (p = 0.07) age-related differences in Breslow thickness of primary melanomas (20–51 yrs: median = 1.1 mm; 52–66 yrs.: median = 1.2 mm; >67 yrs.: median = 1.8 mm).

### Serum 25(OH)D concentrations are associated with survival of melanoma patients

**Figure 3:**
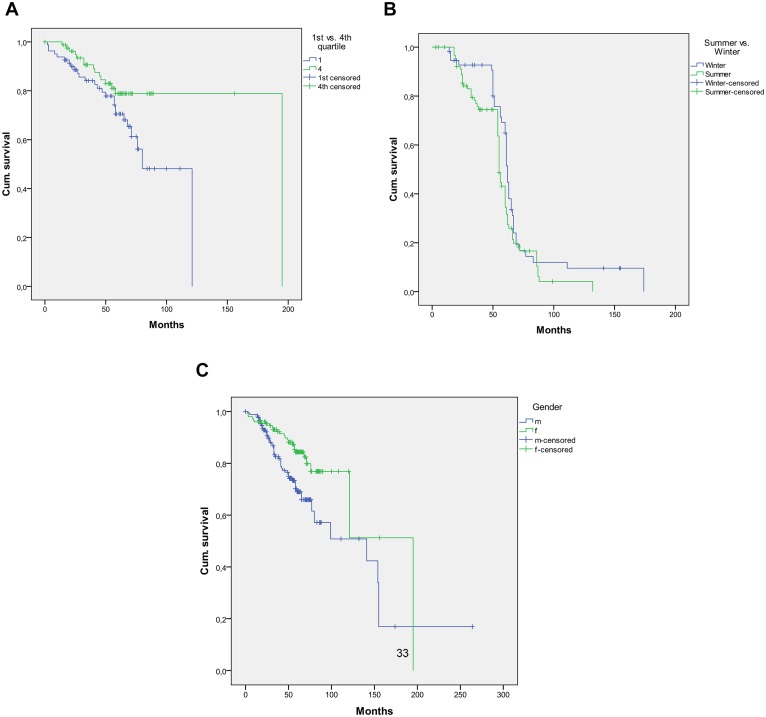
Serum 25(OH)D concentrations are associated with survival of melanoma patients. When dividing the cohort of melanoma patients according to their serum 25(OH)D concentrations into quartiles, a significant difference in survival from time of diagnosis between the 1^st^ (lowest serum 25(OH)D concentrations; 4–9.86 ng/ml) and 4^th^ (highest serum 25(OH)D concentrations; 24.4–59.6 ng/ml) quartiles is found (p = 0.049). Kaplan-Meier analysis shows a median OS of melanoma patients in the 1^st^ quartile (n = 81) of 80 months, while in contrast patients in the 4^th^ quartile (n = 81) have a median OS of 195 months. Age at time of venipuncture has no significant influence on OS (p = 0.2). Tumor stage has a significant influence on OS with p-values<10^−3^ and 0.002 for tumor stages 4 and 3, respectively. This finding may be due to a dependence of tumor stage on 25(OH)D level (3a). Comparing the survival of melanoma patients venipunctured in winter (Nov-Jan) (n = 57) and summer (May-Jul) (n = 92) with each other, no significant difference is observed. The median survival in the winter cohort is 62 months as compared to 57 months in the summer cohort (n = 149; p = 0.056) (3b). The female gender (195 month, n = 150) has a significant longer OS compared to the male gender (141 month, n = 174) (p = 0.003) (3c).

When dividing the cohort of melanoma patients according to their serum 25(OH)D concentrations into quartiles, a significant difference in survival between the 1^st^ (lowest serum 25(OH)D concentrations; 4–9.86 ng/ml) and 4^th^ (highest serum 25(OH)D concentrations; 24.4–59.6 ng/ml) quartiles was found (p = 0.049). Kaplan-Meier analysis showed a median overall survival of melanoma patients in the 1^st^ quartile (n = 81) of 80 months, while in contrast patients in the 4^th^ quartile (n = 81) had a median overall survival of 195 months (Fig. 3a). Age at time of venipuncture has no significant influence on OS (p = 0.2). Tumor stage has a significant influence on OS with p-values <10^−3^ and 0.002 for tumor stages 4 and 3, respectively. This finding may be due to a dependence of tumor stage on 25(OH)D level.

### Season of venipuncture is not associated with survival in melanoma patients

Comparing the time of survival of melanoma patients venipunctured in winter (Nov-Jan) (n = 57) and summer (May-Jul) (n = 92) with each other, no significant difference was observed. The median survival in the winter cohort was 62 months as compared to 57 months in the summer cohort (n = 149; p = 0.056) (Fig. 3b).

### Gender is associated with survival in melanoma patients

The female gender (n = 130) had a significant longer overall survival compared to the male gender (n = 145). In mean, the males survived 71 months in comparison to the females that survived 82 months (p = 0.003) (Fig. 3c).

## Discussion

Even though there is still no agreement of a threshold concentration of serum 25(OH)D for vitamin D deficiency, many experts in the field define it by serum concentrations of 25(OH)D <20 ng/ml [25]. According to this definition, we here report a high prevalence of vitamin D deficiency both in melanoma patients (63%) and in healthy controls (63%) in the German population. Our results are in agreement with other reports published in the literature, reporting a high prevalence of vitamin D deficiency in Germany [34]. Interestingly, median serum 25(OH)D concentrations were moderately but significantly lower (p = 0.004) in melanoma patients (13.6 ng/ml; n = 127) as compared to controls (15.6 ng/ml; n = 141). However, this complex topic requires careful consideration of multiple factors simultaneously. Therefore, we performed additional statistical tests, including age and time of blood draw in multiple analyses and ANOVA, that confirmed our results. Considering 25(OH)D as dependent and sex, age, season of blood draw as well as group as independent variables, the adjusted group effect resulted in a p-value of 0,012. It has to be noted that, although the difference between median concentrations of 13.6 in melanoma patients versus 15.6 in controls is statistically significant, it may be of limited physiologic significance. Moreover, this small difference is within the error of the assay.

Sun avoidance after a diagnosis of malignant melanoma is a common phenomenon, and our present study adds to the growing body of evidence that melanoma patients have suboptimal concentrations of serum 25(OH)D [32]. In a small pilot study, we had failed previously to show lower levels of 25(OH)D in melanoma patients as compared to healthy individuals [35], however these results are likely due to the fact that we analyzed in that study only patients with relatively thin melanomas. Our present study is in agreement with our later follow up study [31], indicating that serum concentrations of 25(OH)D are moderately lower in German melanoma patients as compared to controls. A study reported in 1992 a 5% lower level of 1,25-dihydroxyvitamin D in 23 healthy donors who later went on to develop a melanoma, although this was a non-significant effect [36]. Moreover, we reported previously, that stage IV melanoma patients (metastasized disease) have lower serum concentrations of 25(OH)D as compared to non-metastasized melanoma patients [31]. In our present study, the metastasized melanoma patients collective also showed slightly lower serum concentrations of 25(OH)D as compared to non-metastasized melanoma patients, although these differences were not statistically significant. However, it has to be noted that these findings should be interpreted with care: they represent associations that do not have to be causative for the pathogenesis or progression of melanoma. The possibility must be considered that serum concentrations of 25(OH)D represent a marker for another unknown causal relationship.

Interestingly, we found an association of serum 25(OH)D concentrations, measured at the time of diagnosis, with Breslow thickness of primary melanomas. Vitamin D deficient patients had significantly thicker primary melanomas as compared to patients with a sufficient vitamin D status. These results in German patients are well in line with findings of a prospective cohort study reported in 2009 in another population, showing that melanoma patients with relatively low levels of 25(OH)D have a relatively higher Breslow thickness of their primary melanomas [32]. Moreover, we here report for the first time evidence of an inverse J-shaped curve, indicating that high serum 25(OH)D concentrations (>40 ng/ml) maybe associated with thicker primary melanomas as well. However, it has to be noted that additional clinical data that would be helpful and informative to know were not available for our twelve melanoma patients who had blood levels of 25(OH)D >40 ng/mL. It is generally accepted that it is very difficult to attain a blood level above 40 ng/mL unless a person is exposed to sunlight on a frequent basis and only in the spring and summer and fall. When looking at variations in serum 25(OH)D concentrations throughout the seasons in our melanoma patients (Fig. 2a) it appears that most of the patients were vitamin D deficient but that at each of the seasons several had 25(OH)D blood levels above 40 ng/mL whether it be at the end of the winter or at the end of the summer. This maybe of importance for the interpretation of the so-called inverse J-shaped curve. It may well be that at least some of these individuals were vitamin D deficient and being treated for vitamin D deficiency by their physician. Thus, it can be speculated that these individuals were at a higher risk for having a more aggressive melanoma because of they have been vitamin D deficient; and that only because they were being treated for their vitamin D deficiency at the time of venipuncture their blood level was above 40 ng/mL. According to some studies, the present optimal serum 25(OH)D concentration is approximately 16–24 ng/ml [22], [37]–[39]. However, there is a need for larger, more comprehensive clinical and epidemiological studies [40]. Variations in circulating 25(OH)D concentrations are suggested to be a significantly heritable trait, based on a recent analysis of 1762 elderly men and women in community-based health care [41]. In 2010, common genetic determinants of vitamin D insufficiency have been identified in a genome-wide association study [42]. In that investigation, variants at three loci reached genome-wide significance in discovery cohorts for association with 25-hydroxyvitamin D concentrations, and were confirmed in replication cohorts: 4p12 (overall p = 1.9×10(−109) for rs2282679, in GC); 11q12 (p = 2.1×10(−27) for rs12785878, near DHCR7); and 11p15 (p = 3.3×10(−20) for rs10741657, near CYP2R1) [42]. Participants with a genotype score (combining the three confirmed variants) in the highest quartile were at increased risk of having 25-hydroxyvitamin D concentrations lower than 75 nmol/L (OR 2.47, 95% CI 2.20–2.78, p = 2.3×10(−48)) or lower than 50 nmol/L (1.92, 1.70–2.16, p = 1.0×10(−26)) compared with those in the lowest quartile [42]. It has been concluded that variants near genes involved in cholesterol synthesis, hydroxylation, and vitamin D transport affect vitamin D status and that genetic variation at these loci identifies individuals who have substantially raised risk of vitamin D insufficiency [42].

It is obvious that there has been adaptation to UV-B irradiation such as the skin color, therefore there might be population differences in the optimal 25(OH)D serum concentration. This indicates that additional studies of optimal 25(OH)D serum concentrations for disease prevention are necessary comparing different populations and groups of a population. After those studies, it will be possible to determine what the optimal serum concentration of 25(OH)D for prevention of melanoma and other diseases is.

An important result of this study is the finding, that there is a significant survival benefit (p = 0.049) for patients with higher serum 25(OH)D concentrations (fourth quartile: median survival = 195 months) comparing to patients with lower levels (first quartile: median survival = 80 months). In a prospective cohort study reported in 2009, higher serum concentrations of 25(OH)D were associated in melanoma patients with lower Breslow thickness at diagnosis and were independently protective of relapse and death [32].

Looking at seasonal variations, we were able to observe significant changes in 25(OH)D levels throughout the year (p = 0.000) with expected highest levels in autumn (Aug-Oct) (median = 21.3 ng/ml) like an earlier study showed before [32] and lowest in spring (median = 10.15 ng/ml). The winter results are unexpectedly high due to a peak-increase in January which we cannot explain. Opposite to vitamin 25(OH)D, tumor thickness is indifferent throughout the seasons (p = 0.543), so they seem to have no influence. In our present study, we were unable to show an association of season with overall survival of melanoma patients, as reported by others for other types of cancer [43]: our patients venipunctured in summer (May-Jul) had no significant benefit in overall survival than the ones venipunctured in winter (Nov-Jan).

In our present study, the serum 25(OH)D concentrations in melanoma patients differ statistically significant depending on age. We here report that younger melanoma patients have higher serum 25(OH)D concentrations than older patients. These findings are opposite to results of a previous study in the British population [32] which stated that younger individuals have lower serum 25(OH)D concentrations than older ones. There is typically an increase in overall body fat with ageing, creating a larger distribution volume for the fat-soluble 25(OH)D, and the potential for characterizing patients as vitamin D deficient [44]. Moreover, the capacity of the skin to produce vitamin D decreases with age. Interestingly, younger melanoma patients had thinner primary tumors at time of diagnosis than older patients. These findings are well in line with recent results of a study in the British population [32]. It can be speculated whether the relatively higher vitamin D serum concentrations in the younger population cause this association.

In summary, results of this retrospective study strongly support the hypothesis that the vitamin D status predicts risk and prognosis of melanoma and that melanoma patients have a suboptimal vitamin D status. However, one has to keep in mind that we only report associations, and that a causal relationship has to be confirmed in future studies. Other limitations of our work include the study design (retrospective analyses) and the techniques used (no confirmation of 25(OH)D measurements by mass spectroscopy). Whether normalizing serum 25(OH)D concentrations in these patients improves outcomes will require testing in future clinical trials. Further studies are urgently needed to investigate the role of the vitamin D endocrine system in melanoma.
